# A 2000-year record of fecal biomarkers reveals past herbivore presence and impacts in a catchment in northern Yellowstone National Park, USA

**DOI:** 10.1371/journal.pone.0311950

**Published:** 2024-10-30

**Authors:** John A. F. Wendt, Elena Argiriadis, Cathy Whitlock, Mara Bortolini, Dario Battistel, David B. McWethy

**Affiliations:** 1 Department of Earth Sciences, Montana State University, Bozeman, Montana, United States of America; 2 Department of Environmental Sciences, Informatics and Statistics, Ca’ Foscari University of Venice, Venice, Italy; 3 Institute of Polar Sciences CNR-ISP, Venice, Italy; Kerman University of Medical Sciences, ISLAMIC REPUBLIC OF IRAN

## Abstract

Molecular biomarkers preserved in lake sediments are increasingly used to develop records of past organism occurrence. When linked with traditional paleoecological methods, analysis of molecular biomarkers can yield new insights into the roles of herbivores and other animals in long-term ecosystem dynamics. We sought to determine whether fecal steroids in lake sediments could be used to reconstruct past ungulate use and dominant taxa in a small catchment in northern Yellowstone National Park. To do so, we characterized the fecal steroid profiles of a selection of North American ungulates historically present in the Yellowstone region (bison, elk, moose, mule deer, and pronghorn) and compared them with those of sediments from a small lake in the Yellowstone Northern Range. Analysis of a set of fecal steroids from herbivore dung (Δ^5^-sterols, 5α-stanols, 5β-stanols, epi5β-stanols, stanones, and bile acids) differentiated moose, pronghorn, and mule deer, whereas bison and elk were partially differentiated. Our results show that bison and/or elk were the primary ungulates in the watershed over the past *c*. 2300 years. Fecal steroid influxes reached historically unprecedented levels during the early and middle 20^th^ century, possibly indicating high local use by ungulates. Comparison of fecal steroid influxes with pollen and diatom data suggests that elevated ungulate presence may have contributed to decreased forage taxa (Poaceae, *Artemisia*, and *Salix*), relative to long-term averages, and possibly increased lake production. Our results reflect past change within a single watershed, and extending this approach to a network of sites could provide much-needed information on past herbivore communities, use, and environmental influences in Yellowstone National Park and elsewhere.

## Introduction

The near-extinction of bison (*Bison bison*) in North America in the 19^th^ and 20^th^ centuries ranks among the most dramatic ecological catastrophes in recent times, but little is known about prior herbivore-ecosystem dynamics. The historical record of past distribution and abundance of bison and other large herbivores is limited to the last 200–300 years or less in most portions of western North America [1], and alternative means are required to understand the pre-European ecology of these ungulates and their environments. Much of our information on past bison occurrence comes from fossil vertebrate remains largely recovered from archaeological and paleontological sites [2–4], but these discontinuous records provide limited insights about past variations in habitat use and ungulate community dominance at local scales.

Molecular biomarkers are stable compounds that preserve unique biological signatures and thus provide continuous, high-resolution records of the presence and/or abundance of source organisms and their environment [5]. For example, animal feces contains measurable quantities of steroids that can provide information about an animal’s identity, diet, and intestinal flora [6]. Fecal steroids include Δ^5^-sterols, 5α-stanols, 5β-stanols, epi5β-stanols, stanones, and bile acids. The 5β-stanols and bile acids, in particular, indicate past herbivore presence because herbivores are often their primary environmental source [7, 8]. 5β-stanols are derived from the microbial alteration of precursor sterols (e.g., cholesterol and β-sitosterol) in the intestinal tracts of higher animals (we therefore refer to 5β-stanols as ‘zoostanols’). Primary bile acids (e.g., cholic acid and chenodeoxycholic acid) are synthesized in the liver, stored in the gall bladder, and secreted into the digestive tract, where a fraction is modified by gut bacteria into secondary bile acids (e.g., deoxycholic acid and lithocholic acid) [9, 10]. Anaerobic microbial activity may contribute to low background levels of zoostanols in environmental settings [11, 12]. Bile acids are especially useful for fecal source determination because they are produced exclusively by vertebrates and structure of primary bile acids is genetically encoded and varies across species [10, 13–15]. Zoostanols and secondary bile acids may be directly deposited or transported to lake and wetland sediments, where they can persist for millennia due to their generally high affinity for particulate organic matter [16, 17]. Although analysis of zoostanols and bile acids can potentially provide information about the abundance of source animals (e.g., humans and livestock in high-density settlements as in [18–20]), fecal steroids in sediments have yet to be quantitatively linked to local abundance of wild animals.

Interspecies variability in the relative abundance of zoostanols can facilitate identification of the major contributors of fecal steroids in sediments, as long as prior species distributions are known [21]. Bile acid analysis can further differentiate animal sources because some compounds are exclusively produced by a single, locally present species [6]. Variability among fecal steroid profiles has been used to investigate sources of sewage contamination [22–29] and characterize past agricultural practices in archaeological contexts [6, 18, 21, 30–36], yet zoostanols and bile acids have not been extensively used in continuous lake and wetland sediment cores to reconstruct changes in local paleoherbivore use and dominant taxa over time (but see [37, 38]). Here we use biomarker analysis of modern dung samples and lake sediments from a small watershed in northern Yellowstone National Park to characterize wild herbivore biomarker profiles and infer past herbivore presence and their influence on ecosystem dynamics through time.

**Fig 1:**
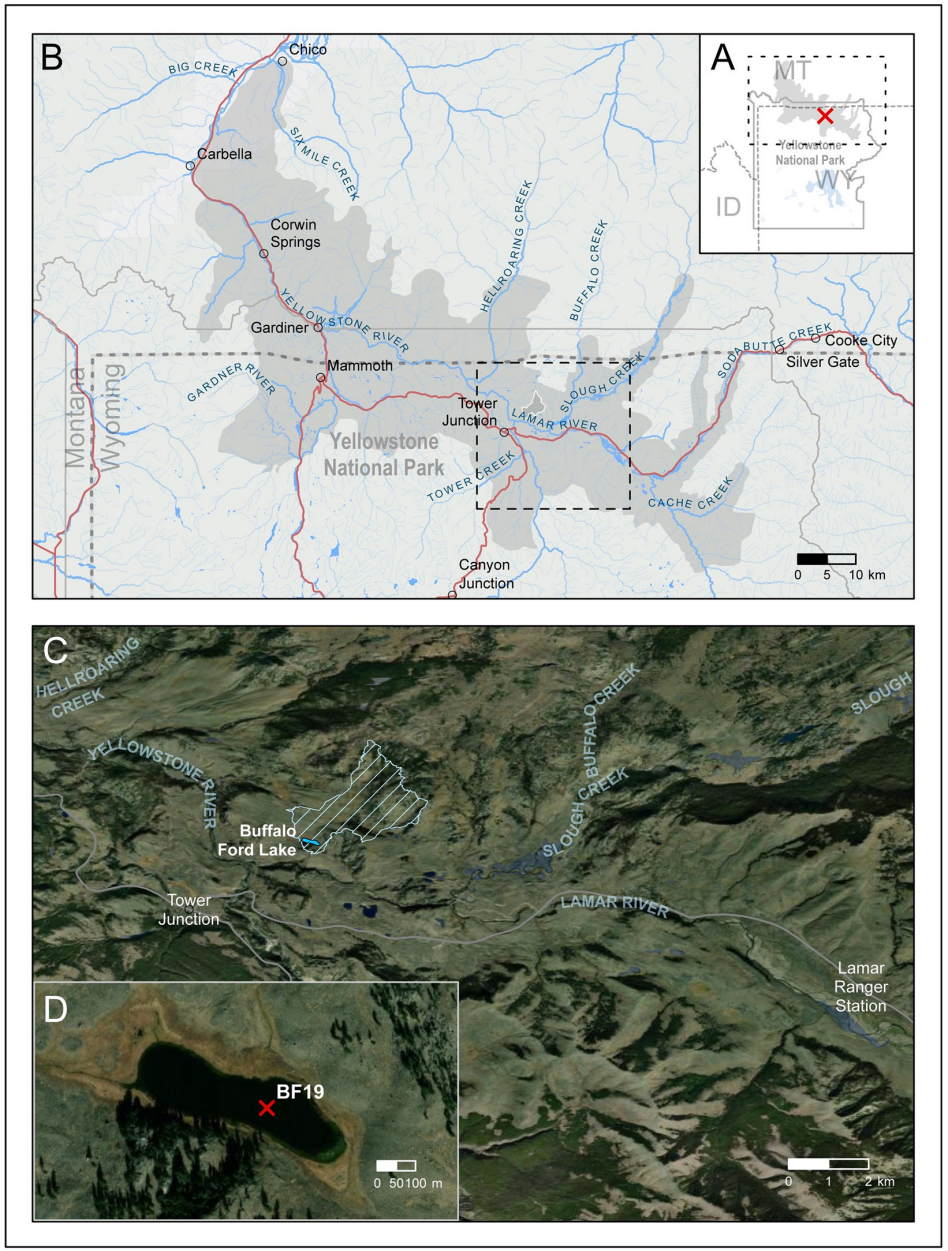
Maps of Yellowstone National Park, the Yellowstone Northern Range, the lower Lamar Valley, and Buffalo Ford Lake. (A) Reference map for Yellowstone National Park, with extent of the Northern Range (gray shading), location of Buffalo Ford Lake (red X), and extent indicator for panel B (black dashed line). (B) Map of the Northern Range (gray shading) with the Buffalo Ford Lake catchment area (white outline), roads (red), rivers and streams (blue), and extent indicator for panel C (dashed black line). (C) Map of the lower Lamar Valley with the Buffalo Ford Lake catchment area (cross-hatched) and Buffalo Ford Lake (blue). (D) Map of Buffalo Ford Lake with location of BF19 core (red X). Basemap sources: Esri, Maxar, Earthstar Geographics, Montana State Library, TomTom, Garmin, SafeGraph, FAO, METI/NASA, USGS, Bureau of Land Management, EPA, NPS, and USFWS.

The Yellowstone Northern Range (Fig 1) refers to the 300,000+ ha landscape of the Lamar, Gardiner, and Yellowstone River valleys in northern Yellowstone National Park and adjoining private and public lands where large free-roaming herds of bison and elk winter [39–41]. The vegetation of the region is primarily grassland and sagebrush steppe, with conifer forests on surrounding highlands, and patches of aspen (*Populus tremuloides*) and willow (*Salix* spp.) in moist lowlands. The Yellowstone Northern Range (hereafter referred to as the Northern Range) provides an ideal setting to test the utility of fecal steroid biomarkers for reconstructing past herbivore use, dominant taxa, and environmental impacts. Today, bison and elk are by far the most abundant ungulates in the Northern Range (S1 Table). Historical population estimates, censuses, and other wildlife management reports over the last 100+ years offer information on past herbivore abundance and distribution that can be used to support interpretations of fecal steroids as proxies for past herbivores. Bison and elk populations in the Northern Range have varied greatly since Yellowstone National Park was established in 1872. The bison population consisted of about 20 individuals at the turn of the 19^th^ century [41] but numbered over 4000 in 2022 [42]. The earliest accepted estimates of Northern Range elk numbers suggest that the population was between 10,000–13,000 in the 1910s and 1920s [43]. The elk population declined to a low count of 3224 in 1968, then peaked at 19,048 in 1994, and numbered 5800 in 2019 [44, 45].

Since Park establishment, managers have struggled to balance protection of bison and elk herds with other resource management goals, including maintenance of habitat integrity [46–49]. The long-running debate over the management of large ungulates in Yellowstone National Park has been challenged by a lack of information on pre-Park occupancy, abundance, and impacts of bison and elk [41, 49–54]. Current understanding of the abundance and community composition of ungulates in the Northern Range is largely based on limited fossil evidence from paleontological and archaeological sites, which confirm past presence of bison and elk in the region and locally [2, 55, 56]. Engstrom et al. [51] sought to identify recent (i.e., past *c*. 100–200 years) herbivore impacts on local vegetation, lake status, and soil stability in the Northern Range through an analysis of the pollen, diatom, and geochemical composition of sediments in small lakes. The study found no strong environmental changes across the region, but the conclusions were hampered by having no way to independently verify local herbivore use through time. In this study, we examine fecal steroid biomarkers in one of the lakes studied by Engstrom et al. to provide this missing piece of information.

Our objectives are to 1) explore the utility of fecal steroids for reconstructing past herbivore presence and dominant taxa in a wild setting; 2) characterize decadal- to millennial-scale changes in herbivore presence and dominant taxa in the Northern Range; and 3) evaluate new and published paleoenvironment data to infer how large herbivores in the Northern Range interacted with their changing environment. To address these objectives, we developed a multi-proxy dataset from Buffalo Ford Lake that builds on Engstrom et al. [51] by describing changes in local herbivore presence, vegetation, fire, and limnology over the last *c*. 2300 years. Our working hypotheses are 1) wild large herbivores can be differentiated by their fecal steroid profiles and 2) fecal steroid influxes of lake sediments reflect local animal use of small catchments.

### Site description

Buffalo Ford Lake (informal name used in Engstrom et al.; 44.934, -110.383; 1921 m elevation) lies in a small (4.3 ha) glacial depression in the lower Lamar Valley of Yellowstone National Park, 1.4 km northeast of the confluence of the Yellowstone and Lamar rivers (Fig 1). The Buffalo Ford Lake catchment (427 ha) and surrounding area supports a variety of large ungulates including bison (*Bison bison*), elk (*Cervus canadensis*), moose (*Alces alces*), pronghorn (*Antilocapra americana*), bighorn sheep (*Ovis canadensis*), and mule deer (*Odocoileus hemionus*).

#### Vegetation

The vegetation in the Buffalo Ford Lake catchment is primarily composed of sagebrush steppe taxa including big sagebrush (*Artemisia tridentata*), rabbitbrush (*Ericameria nauseosa*), and members of Poaceae including Idaho fescue (*Festuca idahoensis*) and Great Basin wild rye (*Leymus cinereus*). Open forest dominated by Douglas-fir (*Pseudotsuga menziesii*), Rocky Mountain juniper (*Juniperus scopulorum*), and limber pine (*Pinus flexilis*) are found on hillslopes around the lake. Lodgepole pine (*Pinus contorta*) grows on rocky outcrops and areas of exposed rhyolite, and Engelmann spruce (*Picea engelmannii*) and subalpine fir (*Abies lasiocarpa*) are present in cool settings and at higher elevation. Small stands of quaking aspen (*Populus tremuloides*) grow in wet seeps and moist settings. Willows (*Salix*) are present in riparian areas. Sedges (Cyperaceae) are found in wet bottom areas, including the margins of Buffalo Ford Lake.

#### Climate

During the period from 1948 to 2005, the Tower Falls weather station (4.5 km south of Buffalo Ford Lake) recorded an average maximum temperature of 11.4°C, an average minimum temperature of -7.1°C, and an average annual precipitation of 42 cm (https://wrcc.dri.edu/cgi-bin/cliMAIN.pl?wy9025). Convective summer storm systems drive summer-wet precipitation patterns in the Northern Range. The wettest three months (May, June, and July) account for 33% of total annual precipitation at Tower Falls. On average, snow begins to accumulate in early November, snow depth peaks in early March, and melt-off ends by early May.

## Materials and methods

### Sediment core collection

A sediment core (BF19) was retrieved in August 2019 from Buffalo Ford Lake (160 cm) from an inflatable raft in the deepest water (5.1 m depth) with a 7.5-cm diameter polycarbonate tube fitted with a piston [57]. The core was collected from the same general location as a prior core in 1987 (BF87) [51]. The BF19 core was stabilized with Zorbitol, sealed, and transported to the Paleoecology Lab at Montana State University where it was split longitudinally and placed in refrigerated storage. Research activities in Yellowstone National Park were conducted under permit YELL-2019-SCI-0009 to CW.

### Sediment core chronology

All statistical analyses were performed in R version 4.1.3 [58] with RStudio [59], unless stated otherwise. The chronology of the BF19 sediment record is based on a Bayesian age-depth model calculated with the IntCal20 calibration curve using the rbacon R package version 2.5.7 (S1 Fig; S2 Table) [60]. The chronology was constructed with two ^14^C samples from BF19, twelve ^210^Pb ages from BF87 [51], and a charcoal peak attributed to the 1988 Yellowstone fires (Table 1). The BF87 ^210^Pb series was anchored to BF19 by assigning the top of BF87, which was deposited in 1987, to 6 cm depth in BF19, which is 1 cm below a charcoal layer attributed to the 1988 Yellowstone fires. This placement is reasonable given the relatively rapid sediment accumulation rates observed in the original BF87 chronology [51]. All ages presented in this paper are calibrated with the IntCal20 curve [61] and given as calendar years before present (cal yr BP), or converted to the Gregorian calendar (BCE/CE). Ages from the 17^th^-21^st^ centuries are given as CE; older ages are given as both cal yr BP and BCE/CE. A sediment accumulation rate (SAR) was calculated for each sampled depth, based on the age-depth model. The formula for SAR is $SAR=\frac{\Delta age}{\Delta depth}$, where Δage is the change in age between two consecutive points (in yr) and Δdepth is the change in depth between the same two points (in cm).

**Table 1:** Calendar ages, AMS radiocarbon dates, and ^210^Pb dates from Buffalo Ford Lake, Wyoming, USA.

Age control/Lab no.	Depth (cm)	^14^C/^210^Pb age ± 2σ error	Cal yr BP (2σ range)	BCE/CE (2σ range)	Material dated
Surface	0	-	-69	2019 CE	-
1988 fire	5	-	-38	1988 CE	Charcoal
210pb-1^a^	6	2 ± 1	-34 (-37, -21)	1984 (1987, 1971) CE	Bulk sediment
210pb-2^a^	9	7 ± 1	-27 (-30, -15)	1977 (1980, 1965) CE	Bulk sediment
210pb-3^a^	11	15 ± 1	-22 (-25, -11)	1972 (1975, 1961) CE	Bulk sediment
210pb-4^a^	15	24 ± 1	-13 (-15, -2)	1963 (1965, 1952) CE	Bulk sediment
210pb-5^a^	18	35 ± 2	0 (-4, 9)	1950 (1954, 1941) CE	Bulk sediment
210pb-6^a^	22	52 ± 2	15 (11, 25)	1935 (1939, 1925) CE	Bulk sediment
210pb-7^a^	25	62 ± 2	27 (21, 36)	1923 (1929, 1914) CE	Bulk sediment
210pb-8^a^	28	71 ± 3	32 (27, 41)	1918 (1923, 1909) CE	Bulk sediment
210pb-9^a^	32	86 ± 8	51 (41, 62)	1899 (1909, 1888) CE	Bulk sediment
210pb-10^a^	35	105 ± 14	65 (46, 89)	1885 (1904, 1861) CE	Bulk sediment
210pb-11^a^	38	127 ± 27	91 (64, 129)	1859 (1886, 1821) CE	Bulk sediment
210pb-12^a^	40	144 ± 49	107 (68, 170)	1843 (1882, 1780) CE	Bulk sediment
210pb-13^a^	43	174 ± 123	148 (91, 246)	1802 (1859, 1704) CE	Bulk sediment
OS-156938^b^	75	785 ± 25	696 (632, 765)	1254 (1318, 1185) CE	Wood
OS-158769^b^	148	2210 ± 20	2188 (2065, 2296)	238 (-115, 346) BCE	Wood

^a 210^Pb ages originally reported in [51]

^b^ Samples measured at NOSAMS Laboratory at Woods Hole Oceanographic Institution

To characterize long-term fire trends and to identify the charcoal layer produced by the 1988 Yellowstone fires, samples of 2 cm^3^ of sediment were taken at contiguous 0.5 cm intervals for the top 11 cm of the core, and at contiguous 1-cm intervals for the remainder of the core for charcoal analysis. Samples were soaked for 24 hours in a 50:50 solution of bleach and 5% sodium hexametaphosphate (NaPO_3_)_6_ to leach color from organic material and deflocculate the sediment. Samples were screened through a 125 μm-mesh sieve. Particles larger than 125 μm were counted under a stereomicroscope based on published studies indicating that particles of this large size typically originate from local fire events [62].

### Biomarker sample preparation

Forty dung samples from five ungulate species (bison, elk, moose, mule deer, and pronghorn) were identified [63] and collected from the CSKT (Confederated Salish and Kootenai Tribes) Bison Range, MT (formerly National Bison Range) and near the Yellowstone National Park boundary in Cooke City, MT and Silver Gate, MT in summer (S3 and S4 Tables). We did not collect dung within Yellowstone National Park boundaries because dung sampling was not an approved activity under our research permit. We selected samples from landscapes that support the same dominant plant taxa as those that grow near our study site, including bunchgrasses (Poaceae), sagebrush (*Artemisia*), and Douglas-fir. Yellowstone National Park and the CSKT Bison Range possess similarly proportioned herds of bison, elk, pronghorn, and mule deer. To reduce the chance of repeat sampling of individuals, dung collection at the CSKT Bison Range was performed by surveying a 10.5-km long by 10-m wide transect and samples were only collected if they had been deposited recently, within *c*. 48 hrs. Pronghorn and mule deer samples were visually confirmed to have come from separate individuals. Bison and elk samples were mainly collected from areas where herds had recently bedded. Dung samples were frozen for storage, air dried, and then freeze-dried. Dung samples were air dried in a running fume hood for 48 hours. Twenty-seven subsamples from BF19 were taken at 2–10 cm intervals, resulting in a mean temporal resolution of 85 years (min: 3 years, max: 209 years; S3 Table). All dung and sediment samples were freeze-dried for 24 hours and then hand-milled and homogenized with a ceramic mortar and pestle. Samples were stored at room temperature in aluminum-foil packets until extraction.

### Biomarker extraction of dung samples

Dung samples were spiked with known quantities of ^13^C-cholesterol (cholesterol-25,26,27-^13^C_3_) and ^13^C-deoxycholic acid (deoxycholic acid-24-^13^C). Dung samples were extracted twice with 20 mL of a 2:1 DCM:MeOH solution in an ultrasonic bath for 15 minutes. Anhydrous Na_2_SO_4_ was added to the extract and a 5 mL aliquot was purified via Pasteur pipette column chromatography with florisil.

### Biomarker extraction of lake-sediment samples

Extraction of biomarkers from lake-sediment samples was performed with an ASE 350 (Accelerated Solvent Extractor, *Dionex Thermo Fisher Scientific*) equipped with 22 mL stainless steel extraction cells. Each sample was mixed with diatomaceous earth and a spike with a known quantity of the ^13^C-labeled internal standards. Extraction was performed twice with a 9:1 DCM:MeOH solution at 100 °C and 1500 psi.

Cleanup and fractionation of dung and sediment samples were performed according to Birk et al. [64], with minor modifications. Extracts were dried under a nitrogen flow and saponified with 3.5 mL of 0.7 M KOH in MeOH (10–14 hours at room temperature), then 10 mL of ultrapure water were added. The neutral fraction (containing sterols, stanols, and stanones) was extracted three times with 15 mL of chloroform, then the sample was acidified with 6 M HCl until pH ≤ 2. An acidic fraction (containing bile acids) was obtained by extracting three times with 15 mL of chloroform.

The neutral fraction was concentrated and purified on neutral silica following the methods described in Battistel et al. [65], dried, remobilized with 100 μL of DCM and derivatized with 100 μL of BSTFA + 1% TCMS, transferred to GC vials and heated to 70°C for 1 hour, cooled to room temperature, and analyzed with GC-MS after 24 hours.

The acidic fraction was dried and methylated with 2 mL of 1.25 M HCl in methanol at 80°C for 2 hours, purified following Birk et al. [64], dried and redissolved in 50 μL of toluene, then derivatized with 100 μL of BSTFA + 1% TMCS, transferred to GC vials and heated to 80°C for 1 hour, cooled to room temperature, and analyzed by a GC-MS (gas chromatograph–mass spectrometer).

### GC–MS analysis

All analyses were performed using an Agilent Technologies 7890 GC system coupled to a 5975C MSD and equipped with a HP-5ms 60 m capillary column (0.25 mm I.D.; 0.25 μm film thickness; Agilent Technologies).

Sterols were separated using the following chromatographic run: 150°C (1 min), 30°C min^-1^ to 220°C (0 min), 0.7°C min^-1^ to 275°C (0 min), 10°C min^-1^ to 300°C (5 min), 10 min at 315°C (post run); helium flow 1 mL min^-1^. Injector and transfer line temperatures were 290°C and 300°C, respectively.

For bile acids, the following chromatographic method was used: 40°C (1 min), 20°C min^-1^ to 230°C (0 min), 2°C min^-1^ to 300°C (20 min), 10 min at 315°C (post run); helium flow 1 mL min^-1^. Injector: 280°C; transfer line: 300°C. For both methods, source temperature was 230°C and quadrupole temperature was 150°C.

### Fecal steroid nomenclature

The fecal stanols measured were coprostanol (5β-cholestan-3β-ol), epicoprostanol (5β-cholestan-3α-ol), cholesterol (cholest-5-en-3β-ol), cholestanol (5α-cholestan-3β-ol), cholestanone (5α-cholestane-3-one), 24-ethylcoprostanol (24-ethyl 5β-cholestan-3β-ol), 24-ethylepicoprostanol (24-ethyl 5β-cholestan-3α-ol), campesterol (campest-5-en-3β-ol), stigmasterol (stigmasta-5,22-dien-3β-ol), β-sitosterol (stigmast-5-en-3β-ol), ergosterol (3β-ergosta-5,7,22-trien-3-ol), and stigmastanol (5α-stigmastan-3β-ol). Bile acids analyzed were chenodeoxycholic acid, deoxycholic acid, hyodeoxycholic acid, lithocholic acid, and ursodeoxycholic acid. Refer to S5 Table for additional compound information.

### Statistical analysis of biomarker profiles

Statistical analysis of fecal stanols followed Harrault et al. [21]. Steroid values of dung and sediment samples were transformed from concentrations to relative abundances of (i) sterols, stanols, and stanones and (ii) bile acids to account for variability among samples. Principal component analysis (PCA) was performed on the relative abundances of each compound in dung samples. Chenodeoxycholic acid and ursodeoxycholic acid were excluded because they were not detected in nearly all dung samples.

This was followed by analysis of the relative proportions of zoostanols, which are predominantly produced in the digestive tract of animals, while other fecal steroids have other environmental sources. We used hierarchical clustering on principal components (HCPC) based on all four principal components, with Euclidean distance and Ward’s method. Lake-sediment samples were added to the PCA as supplementary individuals to assess the similarity of sediment zoostanol profiles to those of the dung. These analyses were performed using the PCA and HCPC functions from the FactoMineR R package [66].

### Lamar Cave fossil record and chronology

The Lamar Cave fossil record consists of multiple sedimentary strata that have been previously analyzed and radiocarbon dated [55, 67], and thus provides an independent record of past presence of ungulate species in the lower Lamar Valley, 2.5 km from Buffalo Ford Lake. The chronology of the Lamar Cave fossil record was updated by converting radiocarbon dates from Hadly [67] to cal yr BP with the most recent calibration curve (Intcal20) with rbacon [60]. Minimum number of individuals (MNI) was used to infer presence or absence of a species within a given stratigraphic level (absence: MNI = 0, presence: MNI > = 1).

### Herbivore population and biomass

Population records for bison (1877–2015 CE) and elk (1911–2015 CE) in the Northern Range were compiled from multiple literature sources [41, 43, 44, 68–70]. Elk population estimates from before the start of aerial surveys in 1952 are less reliable but included here as general estimates. Populations were converted to biomass based on average body masses of 665 kg for bison [71] and 235 kg for elk [72].

We performed tests for association between total elk and bison biomass and biomarker levels (concentration and influx of total zoostanols and lithocholic acid) at Buffalo Ford Lake for two time periods (1920–1970 and 1971–2020) using Pearson’s correlation coefficient. These periods define distinct management regimes that influenced spatiotemporal distribution of elk and bison. Between 1907–1951 elk and bison herds were provided winter hay in the lower Lamar Valley and bison were discouraged from migrating further down valley until the mid-1970s [40, 43]. After 1969, managers began to implement a policy of natural regulation for Northern Range ungulates. Culling ceased, and elk and bison began to expand to new winter range, especially in response to severe winter conditions 1975–1976 [40, 73].

### Pollen analysis

Samples of 1 cm^3^ of sediment were taken at 4–8 cm intervals for pollen analysis resulting in a mean temporal resolution of 101 years (min: 9 years, max: 168 years). The samples were processed according to methods outlined in Bennett and Willis [74]. Each sample was spiked with a known quantity of *Lycopodium* spores to calculate pollen concentration (grains cm^-3^) and pollen accumulation rate (grains cm^-2^ yr^-1^). Pollen residues were mounted in silicone oil and examined at 400-1000x magnification. A minimum of 300 terrestrial pollen grains and spores were identified to the lowest taxonomic level possible on each slide and tallied. Terrestrial taxa were converted to percentages based on the sum of terrestrial pollen and spores. Aquatic pollen counts were converted to percentages relative to the sum of all pollen and spores. Note that Cyperaceae was classified as an aquatic taxon due to its prevalence in surrounding wetlands.

*Pinus* pollen grains with intact distal membranes were identified to subgenus. *Pinus* subg. *Strobus* (haploxylon type, verrucate distal membrane) includes *P*. *albicaulis* and *P*. *flexilis*, which both grow in northern Yellowstone National Park. *Pinus* subg. *Pinus* (diploxylon type, psilate distal membrane) primarily represents *P*. *contorta* in the area. Grains of Cupressaceae are attributed to *Juniperus*-type because *J*. *scopulorum*, *J*. *horizontalis*, and *J*. *communis* are present in the region.

The relative abundance of dominant functional groups (shrubs and grasses versus evergreen conifers) was calculated with pollen percentages as $\frac{Artemisia\;+\;Poaceae}{Pinus\;+\;Pseudotsuga}$. The pollen diagram was created with the rioja R package [75]. High ratio values indicate more open vegetation and low ratio values indicate more forested vegetation.

### Statistical charcoal analysis

Statistical analysis of the Buffalo Ford Lake charcoal record was performed with CharAnalysis software (https://sites.google.com/site/charanalysis) for MatLab following the methods of Higuera et al. [76]. Charcoal counts were converted to charcoal accumulation rate (CHAR; particles cm^-2^ yr^-1^), which was interpreted as an indicator of fire activity or biomass burned. A 500-yr lowess smoother, robust to outliers, was used to characterize long-term background charcoal accumulation rates (BCHAR) [77]. Charcoal peaks were identified as significant fire episodes (a single or sequence of fires within the time span of the sample) if they exceeded the 99^th^ percentile of the local CHAR noise distribution as defined by a Gaussian mixture model.

## Results

### Chronology

The extrapolated age-depth model gives a basal core age of 2290 cal yr BP (2σ 2439–2156 cal yr BP). The median sediment accumulation rate was stable at 0.05–0.07 cm yr^-1^ from the base of the core to *c*. 100 cal yr BP (1850 CE). Sediment accumulation rate sharply increased in the late 19^th^ century and subsequently peaked with an increase to 0.33–0.50 cm yr^-1^ *c*. 1920s CE, stabilized at 0.25 cm yr^-1^ between *c*. 1930–1960 CE, and then rose to 0.50 cm yr^-1^ again from the 1960s CE to the mid-1980s CE. The most recent sediment accumulation rate of 0.17 cm yr^-1^ is lower than the 20^th^ century average but remains elevated relative to the pre-100 cal yr BP (1850 CE) median.

### Lithology

The BF19 sediment core consisted entirely of brown, organic-rich mud, similar to the description in Engstrom et al. [51], which noted that the BF87 core consisted of 44% organic content, 39% inorganic components, and 17% carbonates by dry mass.

### Fecal steroids

Deoxycholic acid was the most prevalent secondary bile acid in the dung of all species analyzed (bison [58%], elk [51%], moose [74%], mule deer [86%], and pronghorn [78%]). Elk dung also contained lithocholic acid (49%), moose feces also contained lithocholic acid (21%) and hyodeoxycholic acid (4%), and mule deer feces also contained lithocholic acid (14%). Elk and mule deer dung did not contain detectable levels of hyodeoxycholic acid. Bison dung was distinguished from other species by relatively high concentrations of hyodeoxycholic acid (11%), while chenodeoxycholic acid was only detected in a single bison dung sample. Ursodeoxycholic acid was not detected in the samples.

**Fig 2:**
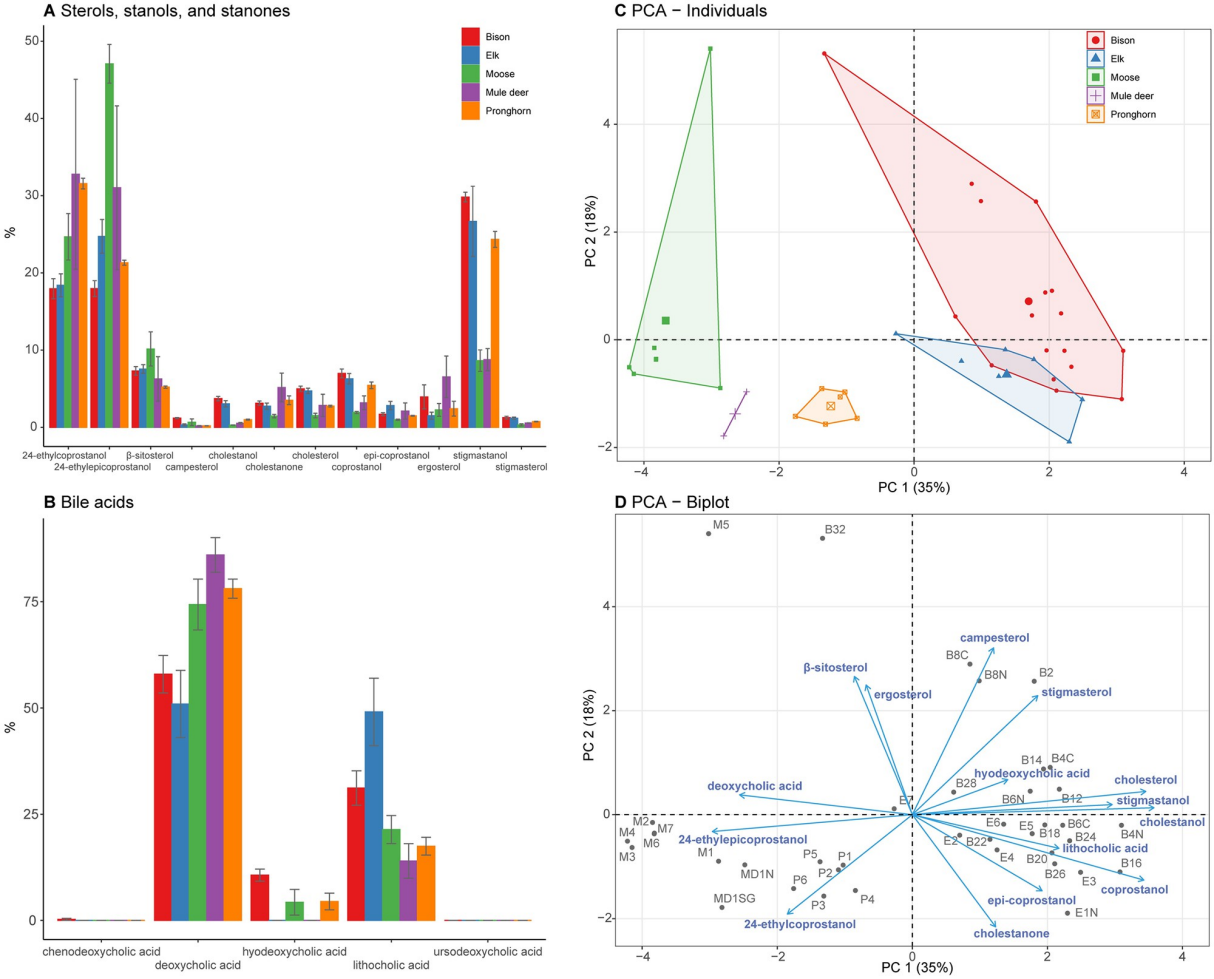
Characterization of fecal steroid profiles bison, elk, moose, mule deer, and pronghorn based on the relative abundances of sterols, stanols, and stanones and bile acids in individual dung samples. Distributions of (A) sterols, stanols, and stanones and (B) bile acids in dung samples by species (mean ± SE). (C) Score plot and (D) biplot showing the first two principal components of the fecal steroid profile PCA. Chenodeoxycholic acid, and ursodeoxycholic acid were excluded from principal components analysis. Number of individuals: bison: 18, elk: 7, moose: 7, mule deer: 2, pronghorn: 6. See S5 Table for compound information. See S3 & S4 Tables for individual sample information and fecal steroid distributions.

Multivariate analysis of fecal steroids indicates that fecal steroid profiles of bison, elk, moose, mule deer, and pronghorn are distinct (Fig 2), and allow complete differentiation of moose, mule deer, and pronghorn and partial differentiation of bison and elk (Fig 2C). However, many fecal steroids have significant non-animal environmental sources, so it is not appropriate to use a broad-spectrum analysis of all steroids to identify ungulate contributors to mixed sediments, such as soils or lake sediments [21]. For this reason, our lake-sediment analysis focuses on zoostanols, which are strong indicators of fecal input.

**Fig 3:**
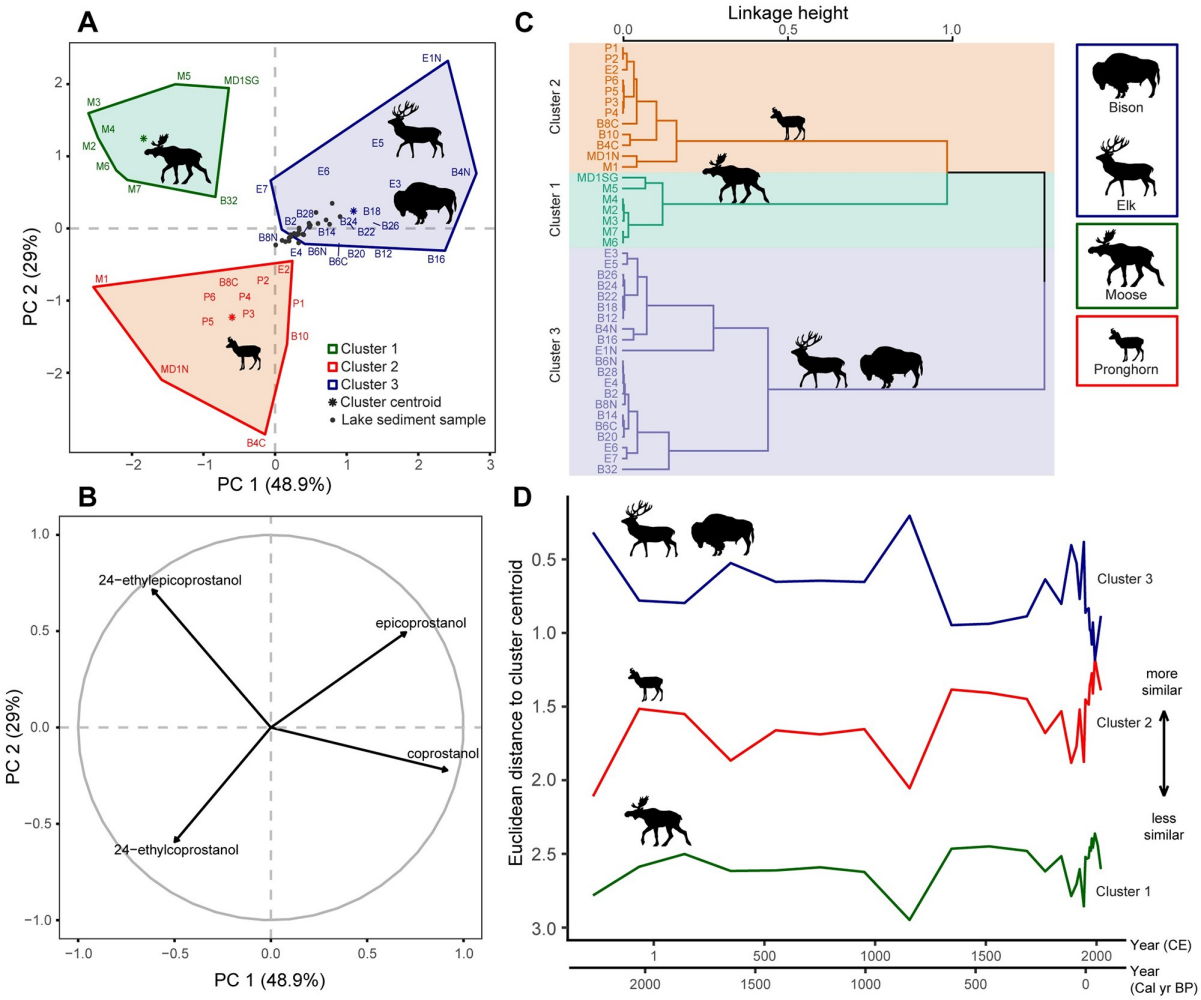
Characterization of 5β-stanol (zoostanol) profiles of sediments from Buffalo Ford Lake in relation to the dung profiles of ungulates. (A) PCA score plot of zoostanols in dung, with lake sediment samples as supplementary observations. Clusters are defined by HCPC (panel C). (B) PCA correlation circle depicting correlation between the first two principal components based on the relative abundance of zoostanols. (C) HCPC dendrogram of zoostanol distributions for individual ungulate dung samples. (D) Euclidean distance of lake-sediment samples to cluster centroids (from panel A). Shorter distance indicates greater similarity of the zoostanol signatures of lake sediment samples to the centroids of the clusters shown in panel A, and thus is an indicator of dominant taxa. The lake sediment samples, which mostly fall within Cluster 3 (panel A) and are all closest to the centroid of Cluster 3 (panel D), strongly indicate that bison and/or elk were the predominant contributors of fecal matter to Buffalo Ford Lake over the last 2 millennia.

Hierarchical clustering on principal components, based on the relative abundances of zoostanols (5β-stanols: coprostanol, epi-coprostanol, 24-ethylcoprostanol, and 24-ethylepicoprostanol) in dung, differentiated three ungulate groups typified by moose (Fig 3, cluster 1), pronghorn (Fig 3, cluster 2), and bison and elk (Fig 3, cluster 3). Bison and elk were not differentiated by their zoostanol profiles alone. The zoostanol profiles of ungulates form a gradient defined by the proportion of 24-ethylcoprostanol and 24-ethylepicoprostanol versus coprostanol and epi-coprostanol (Fig 3B, PC 1). Moose dung contained the greatest proportions of 24-ethylepicoprostanol. Mule deer and pronghorn dung had relatively high levels of 24-ethylcoprostanol. The dung of bison and elk was higher in coprostanol than mule deer and moose, and elk dung contained relatively high quantities of epi-coprostanol.

Euclidean distance to cluster centroids quantifies the similarity of lake sediment zoostanol signatures to those of modern dung samples (Fig 3A). Plotting similarity over time reveals that the zoostanol signature of the lake-sediment samples most closely resembles that of bison and elk throughout the record (Fig 3D, Cluster 3). Browsers such as pronghorn and mule deer may have contributed modestly to the zoostanol composition of the sediment, with greatest relative contributions around *c*. 2000 CE (Fig 3D, Cluster 2). Moose were not well represented by the lake-sediment zoostanol signature, suggesting limited local presence in the Buffalo Ford catchment over the past two millennia (Fig 3D, Cluster 1).

**Fig 4:**
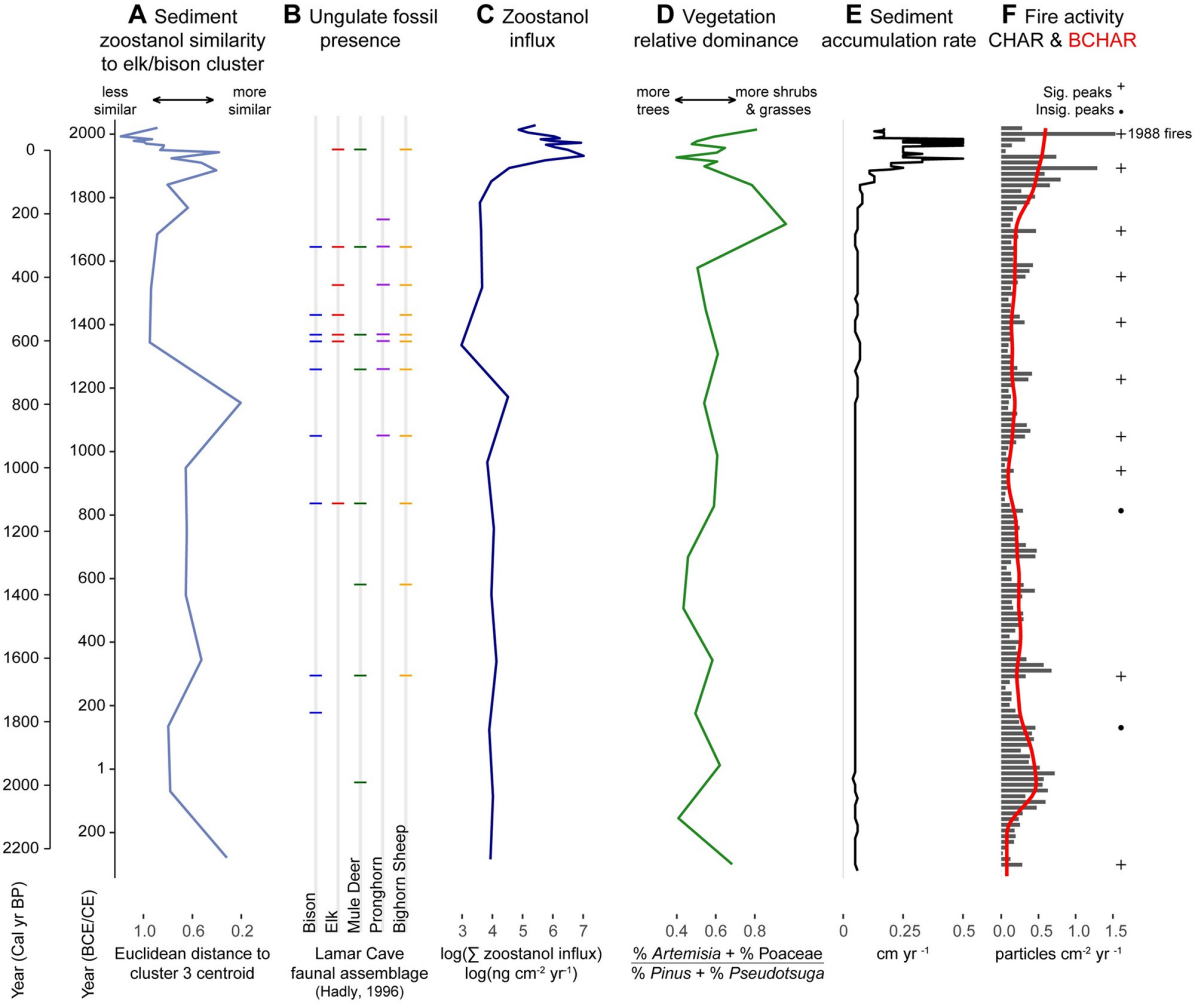
Ungulate community dynamics and associated environmental variables at Buffalo Ford Lake and the lower Lamar Valley during the last *c*. 2300 years. (A) Buffalo Ford Lake sediment similarity to bison and/or elk dung signature, (B) ungulate fossil presence at Lamar Cave, (C) zoostanol influx at Buffalo Ford Lake, (D) pollen percentage ratio, $\frac{Artemisia\;+\;Poaceae}{Pinus\;+\;Pseudotsuga},$ tracking changes in sagebrush steppe and forest cover, (E) sediment accumulation rate (cm yr^-1^), and (F) charcoal accumulation rates and long-term CHAR trends (red line; lowess smoother; window = 500 yr). Statistically significant (+) and insignificant (•) charcoal peaks identify past fire episodes, based on a threshold defined as the 99^th^ percentile of the local CHAR noise distribution.

**Fig 5:**
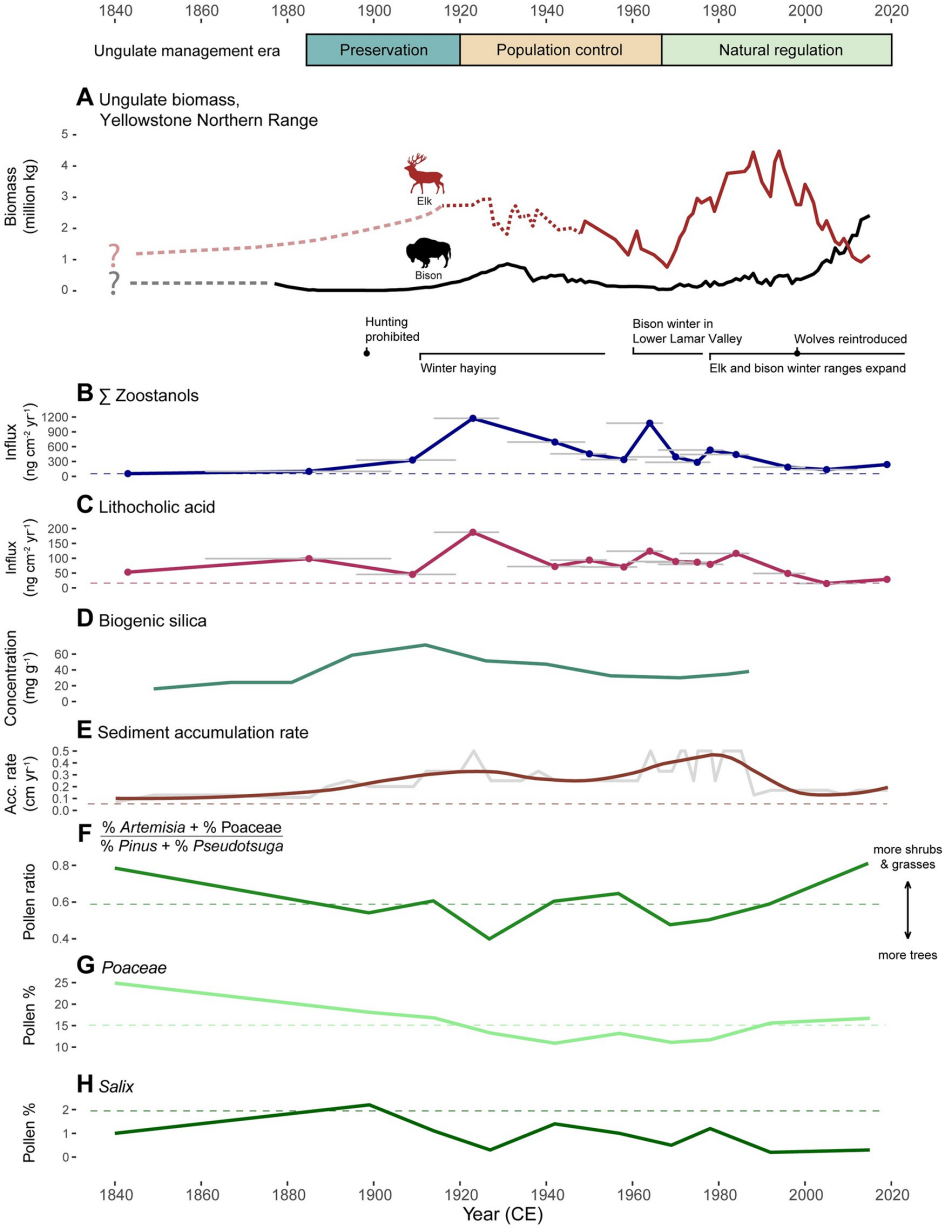
Historical Yellowstone Northern Range bison and elk management regimes in comparison to records of ungulate presence and environmental change at Buffalo Ford Lake. (A) Northern Range elk (red) and bison (black) biomass trends, based on survey records [41, 43, 44, 68–70], with key events noted below. Elk counts before aerial surveys began in 1952 are considered less reliable (dotted line, 1916–1952). Hypothetical pre-survey trends are uncertain and thus indicated with question marks (lighter dashed lines). (B) Influx of total zoostanols (sum of 24-ethylcoprostanol, 24-ethylepicoprostanol, coprostanol, and epicoprostanol) and (C) influx of lithocholic acid. Horizontal gray lines in panels B and C indicate age uncertainty (95% confidence range) associated with sample depths. (D) Biogenic silica concentrations from BF87 [51] show changes in algal production. (E) Sediment accumulation rates based on BF19 & BF87 composite age-depth model (gray line), with loess smoother (light brown), marking periods of increased lake productivity and/or sediment redeposition. (F) Ratio of pollen percentages of steppe-shrub taxa (*Artemisia* + Poaceae) to dominant tree taxa (*Pinus* + *Pseudotsuga*). (G) Poaceae and (H) *Salix* pollen percentages. The dashed horizontal lines in all plots indicate long-term means from 2290 to 100 cal yr BP (340 BCE to 1850 CE).

Influxes of zoostanols and bile acids were generally low and stable throughout the sediment record (BF19) until the mid- to late 19^th^ century, when influxes began to increase dramatically until peaking in the early 20^th^ century (Fig 4 panel C; S2 Fig; S7 and S8 Tables). Fecal steroid influxes remained variable but generally decreased, approaching long-term averages into the 21^st^ century (Fig 5).

Fecal steroid levels were more strongly correlated with elk and bison biomass between 1920–1970 than between 1971–2020, particularly for lithocholic acid concentration, which showed a significant positive correlation 1920–1970 (r = 0.835, p = 0.039; (S3 Fig).

### Pollen

The pollen record indicates that the vegetation around Buffalo Ford Lake experienced only minor fluctuations in relative dominance and limited species turnover during the past *c*. 2300 years (S2 Fig; S6 Table). *Pinus* and *Pseudotsuga* pollen dominate the record with 45–66%, and Poaceae and *Artemisia* together account for 25–43%. *Populus* appears sporadically in the BF19 and BF87 records. *Salix* percentages fluctuate, with a general decline towards present.

Conifer (*Pinus* and *Pseudotsuga*) percentages relative to those of steppe shrubs (*Artemisia*) and grasses (Poaceae) peaked above the long-term average twice during the 20^th^ century (Fig 5). The first peak occurred at *c*. 1930 CE and the second at *c*. 1970 CE. Percentages of *Salix*, an important riparian species and forage for elk, were below the long-term average throughout the 20^th^ century (Fig 5).

### Charcoal

The charcoal record indicates variable fire activity over the last 2300 years at Buffalo Ford Lake (S9 Table). Background charcoal (BCHAR), which indicates biomass burning trends, was relatively high at *c*. 2000 cal yr BP (50 BCE). BCHAR then declined to a minimum at *c*. 1000 cal yr BP (950 CE), and gradually increased over the next 800 years; charcoal accumulation rapidly increased from 200 cal yr BP (1750 CE) to present. Local fires, indicated by significant charcoal peaks, were infrequent between 2300–1000 cal yr BP (350 BCE-950 CE), with only two significant episodes occurring during this period at *c*. 2253 and 1659 cal yr BP (*c*. 303 BCE and 291 CE). Local fires became more frequent from 1000 cal yr BP (950 CE) to present with a mean fire return interval of <100 years (Fig 4).

## Discussion

Analyses of fecal steroid signatures of ungulate species provide new information on herbivore-ecosystem dynamics over the last two millennia in Yellowstone’s Northern Range. The signatures of modern dung samples demonstrate that most Yellowstone ungulate species can be differentiated by their fecal steroid profiles with the exception of bison and elk. Comparison of changes in fecal input based on zoostanol and bile acid influxes (Fig 5B & 5C) with a multi-proxy reconstruction of vegetation (pollen), fire (charcoal), and lake production (sediment accumulation rate and biogenic silica) from a small watershed in northern Yellowstone National Park reveals long-term variations in herbivores and environmental change at the catchment scale. Historical records provide important context for understanding how human management has influenced ungulate habitat use in the lower Lamar Valley (S10 and S11 Tables).

We hypothesize that fecal steroid influxes at Buffalo Ford Lake (Figs 4C, 5B & 5C) indicate animal use of the catchment, especially the lake margin. Datasets documenting historical ungulate density in the area surrounding Buffalo Ford Lake [43, 78] currently lack sufficient temporal coverage to allow rigorous statistical comparison with our fecal steroid data. Correlations of regional elk and bison biomass to sedimentary fecal steroid levels suggest that levels of total zoostanols and lithocholic acid at Buffalo Ford Lake were positively associated with ungulate numbers in the Northern Range between 1920–1970 (S3 Fig), when winter ranges were more spatially restricted than present. Weaker correlations during 1971–2020 (S3 Fig) may reflect multiple management changes implemented in the early 1970s that led to elk and bison winter range expansion and possibly less use of the catchment despite growing populations overall. Thus, in the absence of establishing a quantitative relationship, we find some evidence for a positive relationship between ungulate abundance and fecal steroid levels in lake sediments.

Multivariate analysis indicates that the fecal steroid profile of sediment is most similar to bison and elk across all samples. Therefore, we are confident that bison and/or elk, are the dominant fecal steroid contributors throughout the record. In this case, a categorical (species A or species B) interpretation seems reasonable. We thus interpret dung-sediment similarity (Figs 3D & 4A) as a categorical indicator of dominant taxa, that is, the species that contributed most to the sedimentary fecal steroid signal.

**Fig 6:**
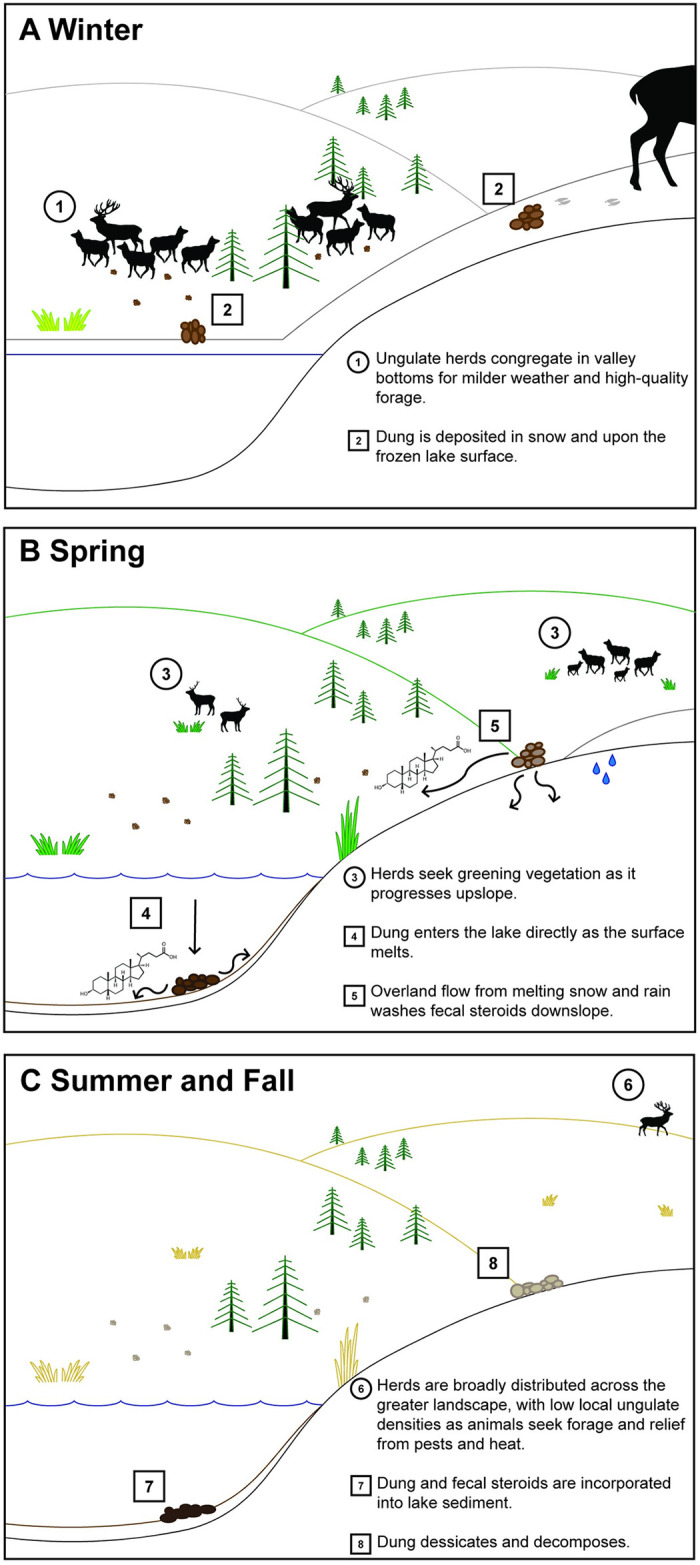
Seasonal patterns of ungulate landscape use in the Yellowstone Northern Range (circles) and hypothesized consequences for the deposition, dispersal, and preservation of fecal steroids (squares) at Buffalo Ford Lake, Wyoming. (A) During winter, ungulate herds concentrate in valley bottoms, leaving dung on frozen lake surfaces and embedded in snow. (B) Warm spring temperatures initiate a wave of greening vegetation that is grazed by ungulate herds, while fecal steroids enter water bodies via thawing ice surfaces and snowmelt runoff. (C) During summer and fall, ungulate herds are widely dispersed across the landscape, often residing at high elevation until snowfall begins. This seasonal ungulate dispersal leads to limited local dung deposition and fecal steroid inputs from transient herds and individuals. Note that bison, although not shown, also move seasonally.

We hypothesize that fecal steroid influxes at Buffalo Ford Lake primarily record the intensity of winter ungulate use within that catchment (Fig 6). During winter, ungulate herds concentrate in valley bottoms to seek milder weather and access to high quality forage (e.g., sedges). Winter conditions also help slow degradation of fecal steroids because lower temperatures reduce microbial activity and snow cover limits damaging solar UV exposure. As temperatures warm in spring, melting snow contributes to overland flow that transports fecal steroids directly into the lake. Dung deposited on the frozen lake surface can rapidly integrate into the lake sediment as the ice thaws in spring. In summer and fall, ungulates are more widely dispersed across the landscape, resulting in more diffuse dung deposition patterns. Fecal steroid influxes thus are likely modified by seasonal weather patterns that influence ungulate distribution, fecal steroid degradation and transportation, and sediment accumulation in the lake. For example, warm temperatures and limited snow cover in winter may result in broader animal dispersal and more rapid degradation of fecal steroids, thereby weakening the ungulate use-fecal steroid influx relationship at the lake. Drier winter conditions might also account for reduced sediment accumulation.

It is probable that fecal steroid signals primarily result from two dispersal vectors: 1) transport from uplands via overland flow; and 2) direct deposition of dung either along lake margins or on the frozen surface in winter. Zoostanols are not easily leached because they are hydrophobic and readily form durable bonds with particles in upper soil layers [16, 17, 79]. In the Northern Range, ungulates often die on frozen lakes during winter, fall through ice, or become mired in floating vegetation mats along lake margins [80]. Decomposing carcasses release substantial quantities of fecal steroids [81]. Further study is needed to quantify the relative fecal steroid contributions of dung from living animals versus carcasses to lake sediments.

Our results show that the dung of many North American ungulate species can be identified by broad-spectrum fecal steroid characterization. To our knowledge, the fecal steroid profiles of bison, elk, pronghorn, and mule deer have not been previously characterized. North American moose dung, like that of Eurasian moose, is characterized by exceptionally high relative levels of 24-ethylcoprostanol and 24-ethylepicoprostanol [21]. Multivariate analysis of sterols, stanones, and stanols with secondary bile acids of sampled species showed complete differentiation of sampled species, except bison and elk (Fig 2). The similarity of bison and elk dung signatures may be due to dietary similarity [82, 83]. Most bison and elk samples were collected from the CSKT Bison Range, where animals are restricted to a smaller area with a lower shrub component than the Northern Range [48, 84]. These conditions may also contribute to higher dietary overlap than we might expect for the Northern Range, where herbivores can access a broader range of options. Bison dung contained hyodeoxycholic acid, which was not detected in elk, and the ratio of lithocholic acid to hyodeoxycholic acid may provide a means to differentiate those taxa in future studies (Fig 2). Hyodeoxycholic acid was not detected in some lake sediment samples, and it is not clear whether those cases represent true bison absences or if levels were simply too low to detect. This issue can be avoided in future studies by extracting fecal steroids from larger lake-sediment samples (ours averaged 0.69 g).

Results indicate that bison and/or elk were the dominant herbivores in the Buffalo Ford Lake area over the past *c*. 2300 years (Fig 3A & 3D). Zoostanol influxes were relatively low and stable in the late 19^th^ century before the establishment of Yellowstone National Park (Fig 4). Zoostanol and bile acid influxes became elevated in the 20^th^ century (Fig 5B & 5C), probably reflecting a local change in ungulate distribution patterns and densities that is observed in regional historical data [43]. This change coincides with a shift in ungulate management from open hunting in the 1860s and 1870s to wildlife protection (public hunting was banned in 1884) and intensive ungulate management (the Lamar Valley was hayed 1907–1951 [41, 43]; Fig 5A).

The fossil assemblage of Lamar Cave, located near Buffalo Ford Lake on the rocky banks of the Lamar River, which provides a discontinuous record of ungulate species presence over the same time span as BF19 (dated to *c*. 3150 cal yr BP, 1200 BCE), includes the remains of bison, elk, mule deer, pronghorn, and bighorn sheep [67]. In contrast, the zoostanol profile from BF19 clearly identifies only the presence of bison and/or elk (Fig 3A). Although the ungulate composition of Lamar Cave may be biased by the behavior of carnivores and scavengers that transported bones to the cave [67], the bone record suggests that elk became more abundant in the last *c*. 600 years (levels 1–9). Low quantities of bison specimens occur in most stratigraphic levels of Lamar Cave, but none were detected within recent (modern-dated) levels. In contrast, white-tailed deer and moose, which are not locally abundant at present according to Park records (S1 Table), were also not present in the Lamar Cave faunal record. Bighorn sheep bones were recovered at Lamar Cave and these animals likely occupied rocky cliffs along the Lamar River and at higher elevations. Bighorn sheep dung was not analyzed in this study, but sheep occasionally use the Buffalo Ford Lake catchment [85]. We assume, however, that they were always less abundant than elk and bison in the open forests and steppe surrounding Buffalo Ford Lake [86].

Comparison of the fecal steroid influxes with historical and paleoenvironmental data suggests some probable environmental impacts of herbivores in the Northern Range, especially during the last two centuries (Fig 5B & 5C; Table 2). It should be noted that distribution patterns and population trends before the 1920s are highly uncertain. Population counts before aerial surveys began in 1952 are considered less accurate compared to recent surveys using modern methods [43].

**Table 2:** Timeline of important events relating to bison and elk in Yellowstone National Park. Adapted from National Research Council et al. [48].

Year/Period	Event
**1860s**	Bison in Paradise Valley exterminated [40]
**1869–1883**	Market hunting reduced populations of ungulates and carnivores [87]
**1870**	Extensive fires in the Northern Range [88]
**1872**	Yellowstone National Park established by Organic Act
**1870–1890**	Major pulse of aspen recruitment [89]
**Late 1870s**	Small summer cattle operation in Lamar Valley [43]
**1880s-1890s**	Small year-round cattle and horse operation in Lamar Valley; hay cut at Slough, Rose, and Soda Bute creeks [43]
**1884**	Public hunting within Yellowstone National Park prohibited by Lacey Act
**1886**	U.S. Cavalry assigned to protect Park; beginning of effective control of hunting [87]
**1890s**	Bison absent from the Northern Range [40]
**1900–1935**	Intensive predator control; wolves extirpated [87]
**1902**	Few bison remained in Yellowstone National Park; population supplemented with outside herds [41]
**1907–1915**	Bison day-herded and fenced at Lamar Ranger Station Buffalo Ranch [41, 43]
**1907–1951**	Winter hay provision in Lamar Valley [40]
**1918**	U.S. National Park Service assumed control of Yellowstone National Park
**1920s**	Increasing concern about overgrazing; active management to control elk population
**1920–1968**	Intensive population control of bison and elk [43, 87]
**1921–1937**	Large quantities of hay fed to bison and elk Slough Creek [43]
**1923–1929**	Elk removed primarily by hunting outside Park boundaries [43]
**1930s-2000s**	Limited aspen recruitment in the Northern Range [43, 89]
**1952**	Beginning of aerial elk population surveys [48]
**1960s-1970s**	Bison winter in Lower Lamar Valley [40, 41]
**1969**	Yellowstone National Park adopts “natural regulation” policy; intensive management of bison and elk ends [90]
**Late 1970s**	Bison and elk winter ranges expand [40, 73]
**1988**	Extensive fires in Yellowstone National Park
**1995**	Reintroduction of wolves
**1995–2015**	Proportion of elk herd wintering in the Northern Range declines [78]

A fire-history reconstruction determined that multiple stands burned in the southern and eastern portions of the Northern Range in 1870 [88]. Journal records also document widespread burning in the summer of 1870 near Tower Junction and attribute these fires to Indigenous burning for the purpose of driving game [91, 92]. The 1870 fire event is the probable source of the significant CHAR peak dated to *c*. 1876 (2σ 1853–1895) that occurred amidst relatively high BCHAR levels that began to increase in the 18^th^ century. Rising BCHAR levels through the 18^th^ and 19^th^ centuries indicate increased natural and/or anthropogenic fires. The 1870 fires, along with reportedly low elk, moose, and beaver populations, relatively moist climate conditions, and wolf presence at the time are thought to have contributed to a major pulse in aspen regeneration in the Northern Range between 1870–1890 [89]. In support of this hypothesis, zoostanol and lithocholic acid influxes at Buffalo Ford Lake are not greatly elevated above the pre-1850 average in the years following the 1870 fires (Fig 5B & 5C).

Two 20^th^ century peaks of zoostanol and lithocholic acid influxes may indicate increased ungulate presence in the Buffalo Ford Lake area (Fig 5B & 5C). The largest peak occurred *c*. 1923 (2σ 1914–1929) when the Northern Range elk population probably numbered between 11,000–13,000 and bison numbers were rising (Fig 5A). Predator suppression, hunting ban enforcement in Yellowstone National Park, and winter hay feeding altered ungulate distributions in the early 20^th^ century (Fig 5) [43]. According to Houston [43], the haying operation along Slough Creek, 3.5 km up valley from Buffalo Ford Lake, most strongly influenced the distribution of bison and elk from 1921 to 1937 when large quantities of hay were provided. Influxes of zoostanols and lithocholic acid reach their record maxima at this time, suggesting that hay provisioning may have contributed to increased ungulate presence in the Buffalo Ford Lake catchment. Declining pollen abundances of forage taxa (e.g., Poaceae and *Salix*) and sagebrush steppe relative to forest taxa between the 1900s and 1940s are consistent with rising elk use of the Buffalo Ford Lake (Fig 5F–5H). Vegetation monitoring data from this time are insufficient to quantify the effects of ungulate population growth on vegetation composition and structure. However, an official report in 1933 expressed concern about the effects of ungulates on the condition of Lamar Valley soils and grassland, noting topsoil erosion, severe overgrazing, and proliferation of grazing-adapted plants [93].

High fecal steroid influxes in the early 20^th^ century are also associated with elevated levels of biogenic silica that peaked *c*. 1912 (2σ 1901–1920; Fig 5D), and a ten-fold increase in sediment accumulation rate at Buffalo Ford Lake *c*. 1923 (2σ 1914–1929; Fig 5E); both are signs of increased lake production [51]. Engstrom *et al*. observed that this phase of increased sediment accumulation likely resulted from in-lake sediment redistribution or increased algal production, rather than increased erosion, because it was not accompanied by a change in sediment composition [51].

Concerns about ungulate overpopulation and habitat degradation in the Northern Range led to a culling program that progressively reduced bison and elk populations between 1931–1967 (Fig 5A) [48, 50]. Fecal steroid influxes generally show a negative trend during the culling period (Fig 5B & 5C), with the notable exception of a peak in zoostanols at *c*. 1964 (2σ 1954–1967; Fig 5B). After winter hay provisioning ceased in 1952, the primary bison winter range shifted to the lower Lamar Valley, where Buffalo Ford Lake is located (Fig 1) [41]. This area served as the herd’s wintering grounds through the 1960s and early 1970s, until the winter of 1975–1976 when severe weather drove bison to seek new foraging sites in the western portion of the Northern Range [40, 41]. High local bison use of the lower Lamar Valley from the late 1950s to the early 1970s may account for the second 20^th^ century decline in sagebrush steppe pollen relative to forest taxa *c*. 1970 at Buffalo Ford Lake (Fig 5F).

Northern Range ungulate populations grew rapidly after 1969 when Yellowstone National Park ended the culling program and initiated a new policy of “natural regulation” [44, 70, 90, 94]. Interestingly, fecal steroid influx trends seem to have decoupled from Northern Range population dynamics at *c*. 1975–1980, when wintering bison and elk populations began to shift down valley (Fig 5). After the winter of 1975–1976, westward bison movements continued annually, resulting in reduced winter use of the lower Lamar Valley [40]. The intensity of winter use by elk likely also decreased at this time, as elk winter range expanded both inside and outside the Park [95]. A minority of the herd began migrations out of Yellowstone National Park after 1975 [43, 73], and in following decades the proportion of elk wintering outside Park boundaries increased after wolf reintroduction and as snow depth increased at higher elevations [78]. Sagebrush steppe pollen subsequently increased, with levels of shrubs and grasses surpassing the long-term mean (Fig 5F). This vegetation shift corresponds with declining fecal steroid influxes (Fig 5B & 5C), which may suggest that reduced local ungulate use led to recovery of upland forage taxa. Increasing sagebrush steppe dominance in the late 20^th^ and early 21^st^ centuries (Fig 5F) may also be related to conifer mortality from the 1988 Yellowstone fires, which burned 40–50% of the Buffalo Ford Lake catchment and much of the surrounding area [96].

Fecal steroid influx was low in Buffalo Ford Lake before the reintroduction of wolves in 1995 (Fig 5B & 5C). Increased pressure from predators may have further reduced bison and/or elk use of the catchment and accounted for the lower steroid levels in the sediments. Elk in the Northern Range often avoid open habitats like the Buffalo Ford Lake catchment during times of day when wolves are active [97, 98], and elk have been shown to reduce herd sizes or move in response to wolf hunting pressure [99, 100]. Therefore, it is possible that elk spent more time near Buffalo Ford Lake when wolves were extirpated (*c*. 1926–1995). On the other hand, elk selected for more open habitat in the winter following wolf reintroduction compared to when wolves were absent [101]. Fecal steroid influxes at Buffalo Ford Lake approached historic lows in the 1990s and have remained low since (Fig 5B & 5C). This observation is consistent with decreasing elk density between the years 1987–1994 and 2005–2011 in the elk count unit that includes the Buffalo Ford Lake catchment (unit #52) [102]. The pollen record suggests limited local willow and aspen abundance despite predator restoration and reduced local ungulate use (Fig 5H and S2 Fig). This is consistent with other observations that show limited willow and aspen recovery following predator restoration [103, 104].

Recent trends in relative ungulate dominance may account for shifts in fecal steroid signatures and quantities in Buffalo Ford Lake sediments. Elk biomass exceeded bison biomass in the Northern Range throughout the 20^th^ century (Fig 5A). In the mid-1990s, elk populations began to decline and bison populations started to grow. Bison biomass surpassed elk biomass in 2010 and continued to rise after. Compared to bison, elk dung tends to have higher levels of epimerized stanols (epicoprostanol and 24-ethylepicoprostanol), relative to the stanol precursors of those compounds (coprostanol and 24-ethylcoprostanol; Figs 2A, 3A & 3B). This difference may explain why the relative prevalence of epimerized stanols decreased in Buffalo Ford Lake as bison became more dominant in recent years (Figs 3D & 4A). In addition, a recent uptick in fecal steroid influxes *c*. 2019 may reflect the recent increase in bison abundance in the Northern Range [105].

Corresponding shifts of fecal steroid influxes and sediment accumulation rates suggest that increased ungulate use may have contributed to nutrient enrichment of Buffalo Ford Lake in the 20^th^ century (Fig 5B, 5C & 5E). This interpretation is supported by diatom and geochemistry records at Slough Creek Lake (44.924, -110.352), which is 2.4 km SE of Buffalo Ford Lake and near historic haying grounds along Slough Creek. These records show two 20^th^ century peaks in biogenic silica, planktonic diatoms, and sediment accumulation rates that are contemporaneous with fecal steroid peaks at Buffalo Ford Lake [51]. Cessation of winter haying in 1952, bison and elk winter range expansions in the late 1970s [40, 73], and reintroduction of wolves in 1995 and natural recovery of cougars [106] may have progressively reduced ungulate use near both Buffalo Ford Lake and Slough Creek Lake. Sediment accumulation rates and fecal steroid influxes at Buffalo Ford Lake have approached lower pre-1850 averages in recent decades (Fig 5B, 5C & 5E).

Our results suggest that interspecific variability of fecal steroids is underpinned by dietary strategies and associated differences in herbivore physiology and anatomy. Dung of browsers, moose, pronghorn, and mule deer [107, 108], is characterized by relatively high proportions of β-sitosterol transformation products, 24-ethylcoprostanol and 24-ethylepicoprostanol, and lower proportions cholesterol transformation products, coprostanol and epi-coprostanol (Fig 2). In contrast, bison and elk, which tend to consume more herbaceous plants [108, 109], are characterized by higher proportions of cholesterol, coprostanol, and epi-coprostanol. Nearly all enteric cholesterol in herbivores is endogenic and not dietary, since plant tissues contain only trace amounts [110]. Sources of enteric cholesterol in ruminants include bile [111] and intestinal cell shedding [112]. Intestinal epithelial cells turn over rapidly (every 4–5 days) [112], and, compared to browsers, grazers possess multiple traits that might increase concentrations of cell-derived cholesterol including longer digestive tracts, with more surface area and longer retention times, after accounting for body mass [113–115]. Longer retention times in grazers may also contribute to greater microbial production of coprostanol and epi-coprostanol. The prevalence of fecal coprostanol and epi-coprostanol seems to result from general physiological and anatomical differences stemming from dietary specialization of grazers and browsers [113, 116]. Further inquiry is needed to discern generality of and the physiological mechanisms underlying the relative abundance of stanols derived from zoosterols versus phytosterols.

### Limitations and opportunities

Fecal steroids at Buffalo Ford Lake are illustrative of the applications and limitations of fecal steroid biomarkers to detect changes in ungulate abundance, dominance, and behavior. Further work is needed to validate the relationship between local animal use and fecal steroid levels in lake sediments. Fecal contributions from non-dominant mammals to sediments are not readily detected using the fecal steroid profiling approach used here. However, some non-dominant species may be detectable with species-specific compounds. For example, ursodeoxycholic acid is a potential indicator of bear presence [117]; it was detected in nearly all sediment samples at Buffalo Ford Lake and not in any ungulate dung tested here. Comprehensive fecal steroid analysis of more animal species will reveal the degree of specificity provided by fecal steroids, especially secondary bile acids.

Although regional population size is an important driver of local ungulate use, large herbivores, including bison and elk, can travel great distances [118, 119] and generally use landscapes unevenly [120–122]. Large herbivores may concentrate in or avoid certain areas in response to predator pressure, forage availability and quality, water distribution, thermal regulation needs, or other factors [101, 123–125]. Additionally, shifting seasonal migration patterns over time can change spatial occupancy and local densities. For example, the Lamar Valley was a primary wintering ground for Yellowstone elk in the 1960s, yet by the early 21^st^ century fewer elk wintered there due to mortality of adult females and calves, and meanwhile bison numbers have increased [118, 126]. Fecal steroids in lake sediments within small catchments are unlikely to match these broad-scale population dynamics and greater success may come from the analysis of multi-site networks designed to capture spatial and temporal variability of mobile herds. Fecal steroid records from multiple catchments are also needed to develop quantitative comparisons with regional animal abundance. Given the region’s wealth of existing lake-based paleoenvironmental data [51, 127–129], detailed information on past and present animal populations [41, 43, 44, 118, 120, 130], and stakeholder interest in conservation informed by paleobiology [49, 131, 132], the Yellowstone Northern Range is an ideal setting to refine interpretations of sedimentary fecal steroid records and develop new records at regional scale.

## Conclusions

Fecal steroid biomarkers in lake sediments are promising tools to help managers and conservationists understand wild ungulate patterns through time. Although written records in the Americas are limited to the past several hundred years, sediments containing lipid biomarkers extend to the Pleistocene and beyond [133, 134]. Analysis of the Buffalo Ford Lake record demonstrates how such records can be used to identify dominant ungulate taxa, presence, and environmental impacts over multiple timescales. The Buffalo Ford Lake record provides important context for understanding ungulate occupancy of a watershed that is consistent with regional records of their management and seasonal patterns of use. Specifically, our results point to two millennia of continuous presence of bison and/or elk and exceptionally high impacts by these ungulates in the 20^th^ century when hunting was banned, predators were suppressed, winter forage was supplemented with hay, and range expansions were actively discouraged.

Whereas other studies have used fecal steroids to identify animal contributors to fecal matter in agricultural settings (e.g., [6, 21]) and to reconstruct local use by humans and domestic livestock over time (e.g., [18, 38]), this study establishes proof of concept for the application of fecal steroids in lake sediments to determine past presence of non-domestic, native herbivores in wild settings. Calibration studies are needed to evaluate the sensitivity of fecal steroids to changes in local herbivore density and seasonal use, and further development of biomarker reference databases will help characterize intra- and interspecies variability and thereby improve differentiation. Additionally, future research comparing fecal steroid biomarkers with other ecosystem proxies in a network of sites can potentially provide key insights into herbivore population dynamics and their ecological impacts through time. Combined analysis of fecal steroid biomarkers with sedimentary ancient DNA (sedaDNA) to determine presence of non-dominant taxa and stable isotopes of strontium (^87^Sr/^88^Sr) and oxygen (δ^18^O) in fossil teeth to reconstruct seasonal patterns of animal movement can reveal more details about the identity and behavior of ancient herbivores. Continued development of fecal steroid records can address critical questions about the historic role of bison, elk, and other ungulates in Yellowstone National Park and other grazed ecosystems.

## Supporting information

S1 Fig Composite age-depth model and sediment accumulation rate for BF19 & BF87. (A) Composite age-depth model for Buffalo Ford Lake based on ^14^C dates from BF19 (blue density curves), ^210^Pb dates from BF87 (green density curves), and the charcoal lens from the 1988 Yellowstone fires using rbacon version 2.5.7 [60]. (B) Sediment accumulation rate over core depth. The dotted red line represents the weighted mean age at a given depth, gray shading and the dotted gray lines represent the distribution of the most likely age-depth model and 95% posterior density intervals.
(TIF)

S2 Fig Multiproxy summary of pollen, fecal biomarker, and charcoal data for BF19. Percentage diagrams of major pollen types and spores, total sum of terrestrial pollen, the ratio of *Artemisia* and Poaceae *to Pinus* and *Pseudotsuga* pollen, local ungulate use based on fecal steroid biomarkers (total zoostanols: sum of 24-ethylcoprostanol, 24-ethylepicoprostanol, coprostanol, and epicoprostanol), and charcoal data (CHAR and BCHAR) with significant and insignificant peaks from Buffalo Ford Lake 2019 sediment core. Curve exaggeration is represented by light shading.
(TIF)

S3 Fig Correlations of Yellowstone Northern Range elk & bison biomass and fecal steroid levels for BF19. Fecal steroid levels are quantified as concentrations (panels A & B) and influxes (panels C & D). Linear models were fitted for the time periods 1920–1970 (blue) and 1971–2020 (red). Pearson correlation test results are reported for both time periods.
(TIF)

S1 Table Recent population estimates for ungulate species in the Yellowstone Northern Range. (XLSX)

S2 Table Composite age-depth model for BF19 & BF87. Median accumulation rate expressed in cm yr^-1^.
(XLSX)

S3 Table Ungulate dung sterol, stanol, and stanone concentrations. Concentrations expressed in μg g^-1^.
(XLSX)

S4 Table Ungulate dung bile acid concentrations. Concentrations expressed in μg g^-1^.
(XLSX)

S5 Table Compound information for analyzed fecal steroids. (XLSX)

S6 Table Buffalo Ford Lake (BF19) pollen percentages. (CSV)

S7 Table Buffalo Ford Lake (BF19) sterol, stanol, and stanone concentrations. Concentrations expressed in ng g^-1^.
(XLSX)

S8 Table Buffalo Ford Lake (BF19) bile acid concentrations. Concentrations expressed in ng g^-1^.
(XLSX)

S9 Table Buffalo Ford Lake (BF19) charcoal counts and concentrations. Concentrations expressed in pieces cm^-3^.
(CSV)

S10 Table Elk population history in the Yellowstone Northern Range, 1916–2015. (XLSX)

S11 Table Bison population history in the Yellowstone Northern Range, 1877–2015. (XLSX)
