# Supplementary figures and images for: A 2000-year record of fecal biomarkers reveals past herbivore presence and impacts in a catchment in northern Yellowstone National Park, USA
Source: PLoS One. 2024 Oct 30;19(10):e0311950. doi: 10.1371/journal.pone.0311950 (PMC11524497; doi:10.1371/journal.pone.0311950)

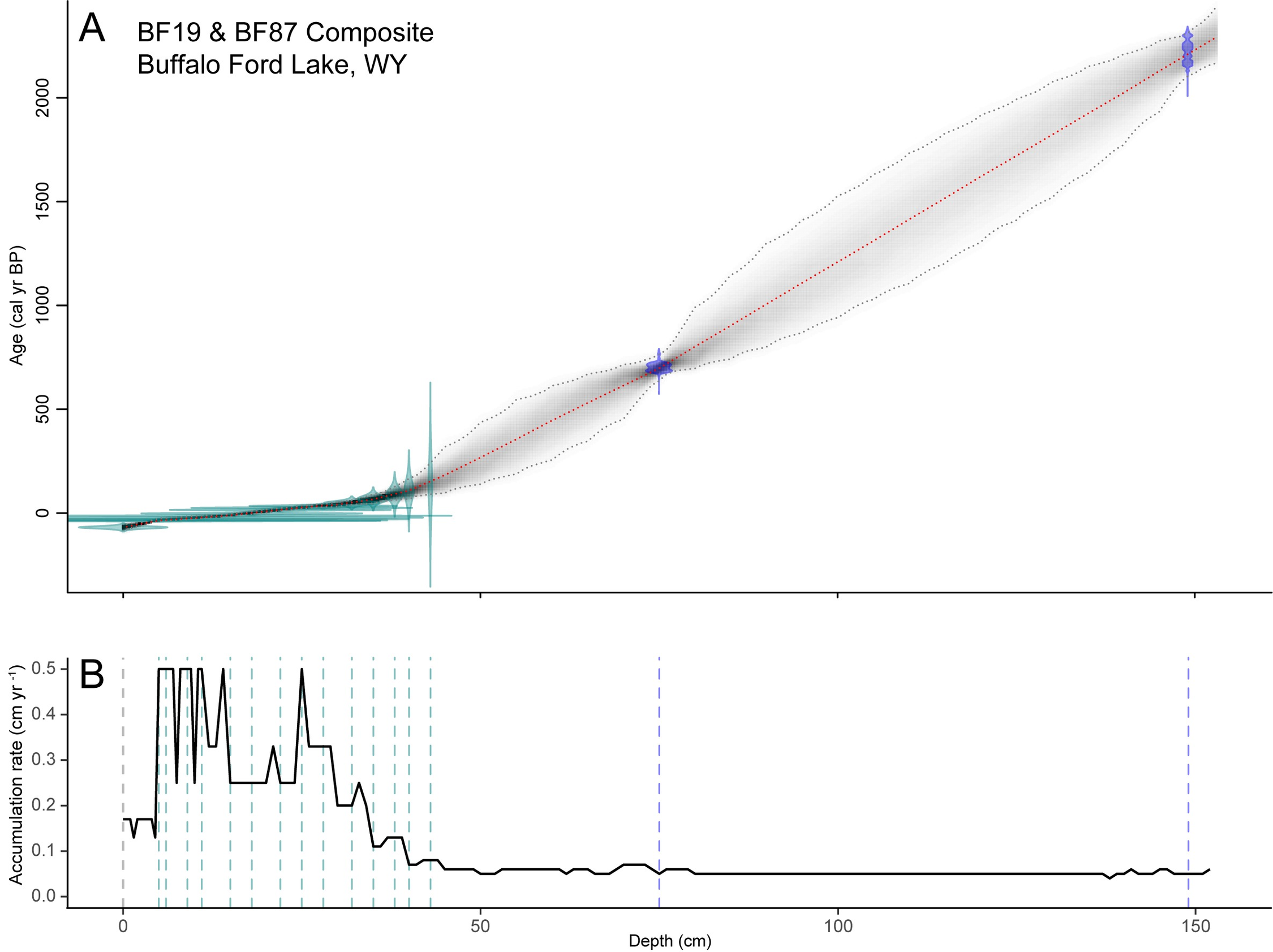

Supplement: S1 Fig — (A) Composite age-depth model for Buffalo Ford Lake based on 14C dates from BF19 (blue density curves), 210Pb dates from BF87 (green density curves), and the charcoal lens from the 1988 Yellowstone fires using rbacon version 2.5.7 [60]. (B) Sediment accumulation rate over core depth. The dotted red line represents the weighted mean age at a given depth, gray shading and the dotted gray lines represent the distribution of the most likely age-depth model and 95% posterior density intervals. (TIF) [file pone.0311950.s001.tif]

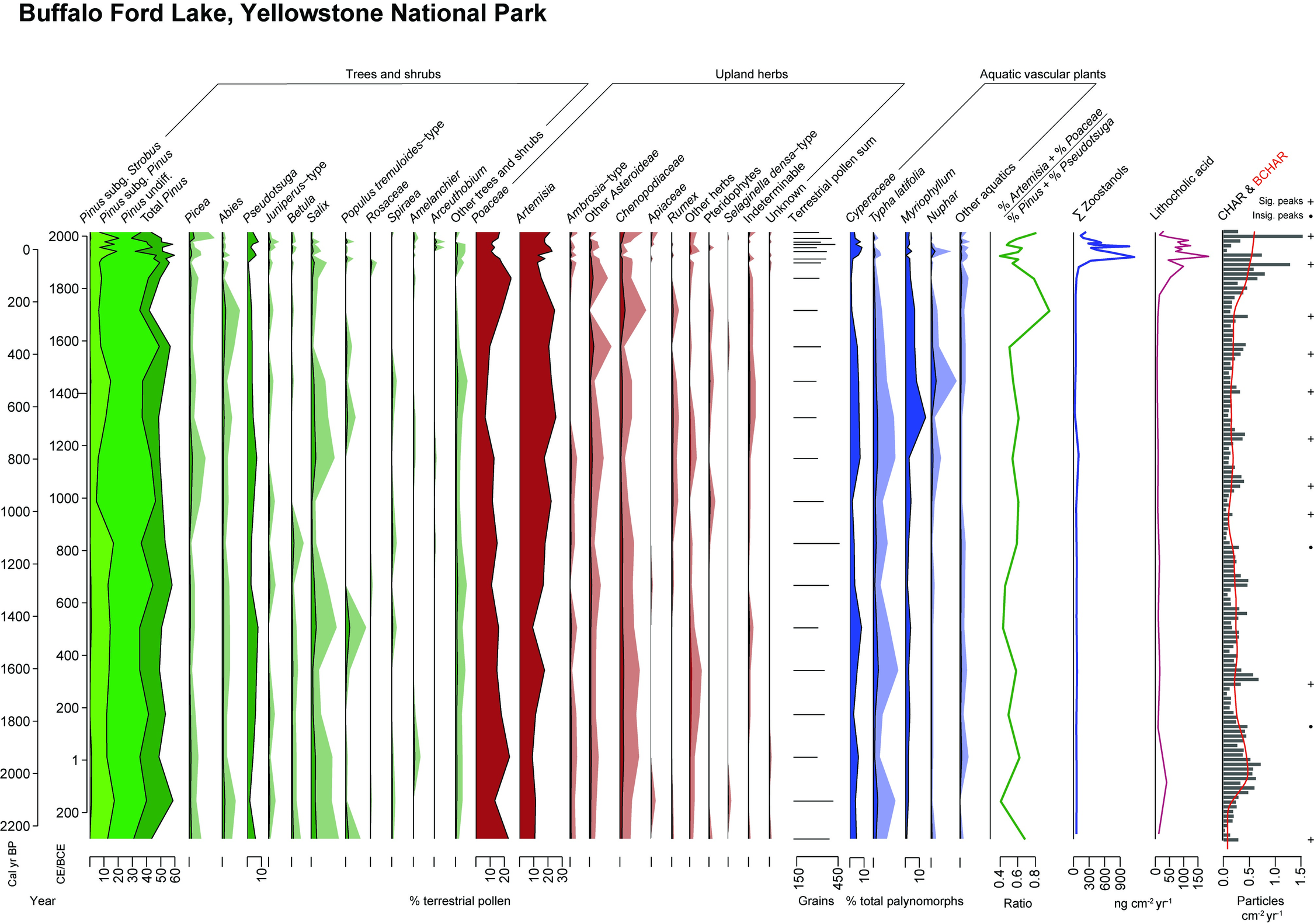

Supplement: S2 Fig — Percentage diagrams of major pollen types and spores, total sum of terrestrial pollen, the ratio of Artemisia and Poaceae to Pinus and Pseudotsuga pollen, local ungulate use based on fecal steroid biomarkers (total zoostanols: sum of 24-ethylcoprostanol, 24-ethylepicoprostanol, coprostanol, and epicoprostanol), and charcoal data (CHAR and BCHAR) with significant and insignificant peaks from Buffalo Ford Lake 2019 sediment core. Curve exaggeration is represented by light shading. (TIF) [file pone.0311950.s002.tif]

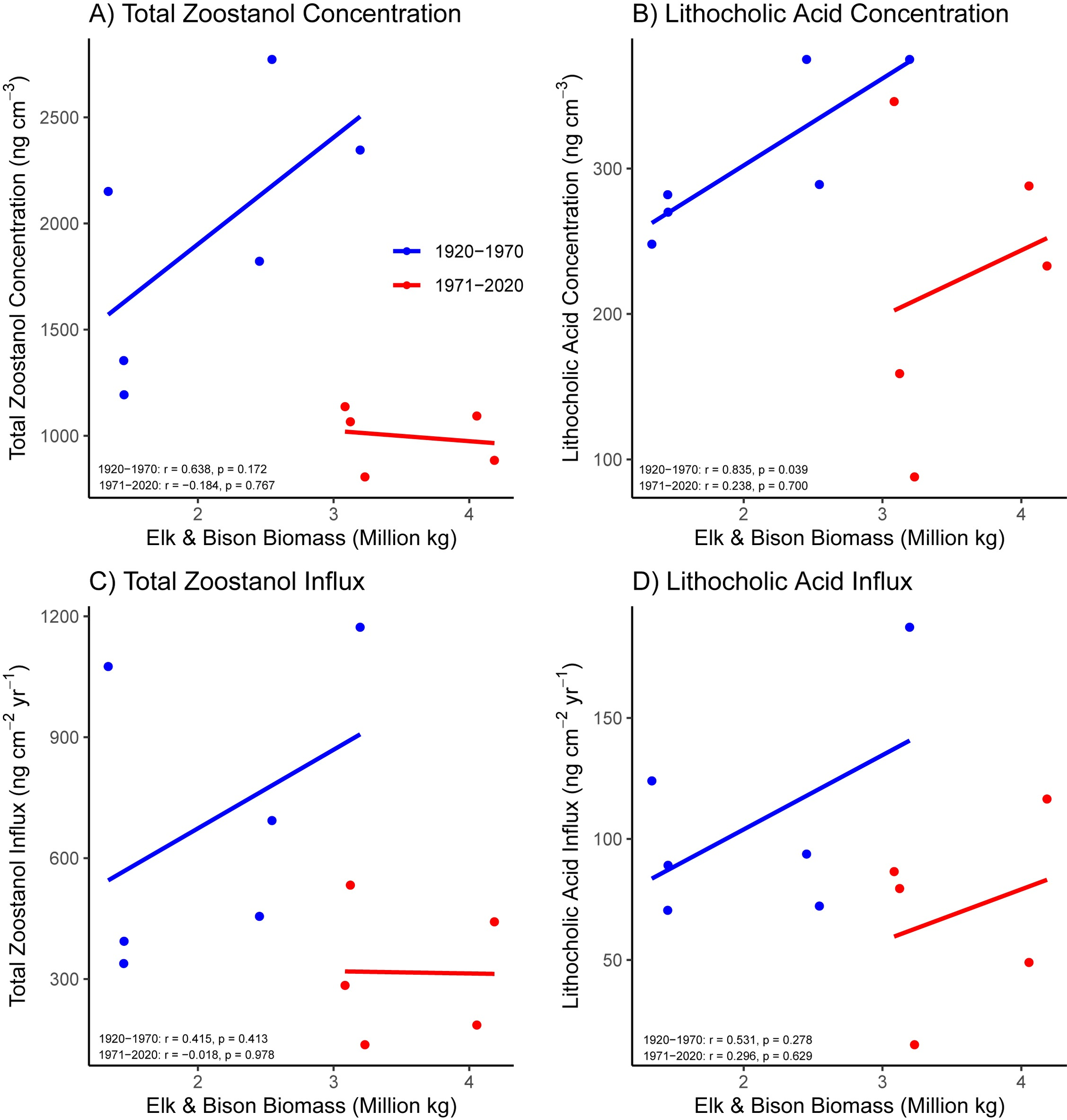

Supplement: S3 Fig — Fecal steroid levels are quantified as concentrations (panels A & B) and influxes (panels C & D). Linear models were fitted for the time periods 1920–1970 (blue) and 1971–2020 (red). Pearson correlation test results are reported for both time periods. (TIF) [file pone.0311950.s003.tif]
